# Upregulation of Na_v_1.6 Mediated by the p38 MAPK Pathway in the Dorsal Root Ganglia Contributes to Cancer-Induced Bone Pain in Rats

**DOI:** 10.3390/cells11213375

**Published:** 2022-10-26

**Authors:** Mingxue Lin, Xiaohui Chen, Shuyan Wu, Pinzhong Chen, Haiyang Wan, Simeng Ma, Na Lin, Yanling Liao, Ting Zheng, Jundan Jiang, Xiaochun Zheng

**Affiliations:** 1 Department of Anesthesiology, Shengli Clinical Medical College of Fujian Medical University, Fujian Provincial Hospital, Fuzhou 350000, China; 2 Department of Anesthesiology, First Affiliated Hospital of Yangtze University, First People’s Hospital of Jingzhou, Jingzhou 434000, China; 3 Fujian Provincial Maternity and Children’s Hospital, Fuzhou 350000, China; 4 Fujian Emergency Medical Center, Fujian Provincial Key Laboratory of Critical Care Medicine, Fuzhou 350000, China

**Keywords:** cancer-induced bone pain, RNA-seq, dorsal root ganglion, Na_v_1.6, MAPK pathway

## Abstract

Cancer-induced bone pain (CIBP) occurs frequently among advanced cancer patients. Voltage-gated sodium channels (VGSCs) have been associated with chronic pain, but how VGSCs function in CIBP is poorly understood. Here, we aimed to investigate the specific role of VGSCs in the dorsal root ganglia (DRGs) in CIBP. A CIBP rat model was generated by the intratibial inoculation of MRMT-1 breast carcinoma cells. Transcriptome sequencing was conducted to assess the gene expression profiles. The expression levels of key genes and differentiated genes related to activated pathways were measured by Western blotting and qPCR. We implanted a catheter intrathecally for the administration of lentivirus and drugs. Then, the changes in the mechanical withdrawal threshold (MWT) were measured. We identified 149 differentially expressed mRNAs (DEmRNAs) in the DRGs of CIBP model rats. The expression of Na_v_1.6, which was among these DEmRNAs, was significantly upregulated. The Kyoto Encyclopedia of Genes and Genomes (KEGG) pathway analysis of the DEmRNAs showed that they were mainly enriched in the mitogen-activated protein kinase (MAPK) pathway. The decrease in MWT induced by bone cancer was attenuated by Na_v_1.6 knockdown. Western blot analysis revealed that a p38 inhibitor decreased the expression of Na_v_1.6 and attenuated pain behavior. Our study shows that the upregulation of Na_v_1.6 expression by p38 MAPK in the DRGs of rats contributes to CIBP.

## 1. Introduction

Clinically, many tumors, such as breast, lung, prostate, and kidney cancer, have a strong predilection to metastasize to bone [1]. Up to 75% of patients with bone metastasis tumors have moderate to severe pain, which has a serious impact on the patients’ mood and quality of life [2]_._ Cancer-induced bone pain (CIBP) is a complicated pain condition consisting of basal, spontaneous, or exercise-induced pain [3,4]. Clinically available drug treatment options for CIBP are limited by their inability to sufficiently relieve pain or their intolerable side effects [5,6]. Therefore, effective alleviation of CIBP has become a challenge in the clinic, and further studies of the mechanism underlying CIBP are urgently needed.

The dorsal root ganglia (DRGs) consist of the cell bodies of primary sensory neurons [7]. The voltage-gated sodium channels (VGSCs) of the DRGs underlie the transduction and propagation of nociceptive signals [8]. Several studies have shown that changes in VGSCs in DRG neurons have a decisive effect on the excitability of DRG neurons, resulting in chronic pain sensations, including neuropathic pain and inflammatory pain [9,10,11]. However, the specific mechanisms by which VGSCs influence CIBP are undefined.

In this study, our purpose was to clarify the specific isoforms of VGSCs that are altered in the DRGs of CIBP model rats. For this purpose, transcriptome sequencing technology was employed to analyze transcriptional alterations in the DRGs. The results showed that the Na_v_1.6 mRNA expression was significantly upregulated in rats that received intratibial injection of breast cancer cells.

Na_v_1.6, which is encoded by sodium voltage-gated channel α subunit 8 (*Scn8a*), is a VGSC subtype that is widely expressed in the nervous system. Na_v_1.6 produces a persistent and resurgent current, in addition to larger transient, fast-inactivating sodium currents, as the basis for action potentials [12]. Indeed, in the peripheral nervous system, a mutation in Na_v_1.6 leads to trigeminal neuralgia by lowering the current threshold and increasing the frequency of the evoked action potentials in trigeminal ganglion (TRG) neurons [13]. Na_v_1.6 is also one of the major channels in DRGs. In a rat neuropathic pain model, mechanical hyperalgesia was effectively alleviated by local downregulation of Na_v_1.6 in the DRGs [14]. Increasing evidence indicates that Na_v_1.6 plays an important role in chronic pain, but the role of Na_v_1.6 in CIBP has not been clarified and needs to be further researched.

The expression of VGSCs is modulated by different signaling pathways. In vitro, it was found that activation of the NK-1 receptor can potentiate Na_v_1.8 sodium currents in DRGs mediated by the PKCepsilon-dependent signaling pathway [15]. Kao et al. also showed that CC chemokine ligand 2 can promote the expression of Na_v_1.8 sodium channels in cultured DRGs by activating the PI3K/Akt signaling pathway [16]. However, the upstream regulatory mechanisms of Na_v_1.6 have not been well established. In our study, the mitogen-activated protein kinase (MAPK) signaling is one of the major Kyoto Encyclopedia of Genes and Genomes (KEGG) pathways in which the differentially expressed mRNAs (DEmRNAs) were enriched. MAPK cascades play important roles in transducing extracellular stimuli into intracellular responses and regulating the transcription of target genes that have been implicated in pain diseases. It has been reported that activated p38α phosphorylates Na_v_1.6 in rat brain tissue, leading to a marked decrease in the Na_v_1.6 current. In addition, in vitro, the current density of the TTX-R current in the DRGs, which is mainly induced by Na_v_1.8, is increased in a p-p38-dependent manner after acute treatment with tumor necrosis factor-α. Previous studies have shown that VGSCs may be regulated by MAPKs. However, the effects of MAPKs on Na_v_1.6 channels in the development of CIBP have not been reported.

In our study, we explored the effect of Na_v_1.6 in the DRGs in CIBP and determined whether MAPK pathways are involved in CIBP by regulating Na_v_1.6 expression.

## 2. Materials and Methods

### 2.1. Experimental Animals

Our experiments obtained female Sprague Dawley (SD) rats from Shanghai SLAC Laboratory Animal Co., Ltd., weighing approximately 150–180 g at operation. Five rats were housed per cage under controlled temperature conditions, with a 12 h of light and 12 h of darkness. Water and food were provided to the rats. The procedures described were approved by the Animal Welfare and Ethics Committee of Fujian Medical University and were performed following the National Institutes of Health guidelines.

### 2.2. Establishment of a CIBP Rat Model

MRMT-1 rat breast cancer cells (JENNIO Biological Technology, China) were used to establish a CIBP model. On the experimental day of establishing the model, we used phosphate-buffered saline (PBS) trypsinized with 0.25% trypsin-0.53 mM EDTA to wash MRMT-1 carcinoma cells twice. Then, the cells were centrifuged for 5 min at 350 *g*. We also used Hanks’ balanced salt solution (HBSS) without Ca^2+^, Mg^2+^, or phenol red (Gibco) to wash the pellet twice, and the cells were counted and suspended in an HBSS solution at a density of 3 × 10^7^ cells/mL and were maintained on ice.

The CIBP model was established using a reported method [17,18]. Briefly, all rats were anesthetized by the inhalation of 1–2% isoflurane. The skin was shaved, and the area was disinfected. To expose the tibia, an incision was made on the anterior–medial surface. A hole was made 5 mm below the knee joint with a 24-gauge needle. Then, a microinjection syringe was inserted into the intramedullary cavity at a depth of 1 cm, and 3 × 10^5^ MRMT-1 carcinoma cells in 10 µL of HBSS or 10 µL of HBSS alone were injected. The syringe was removed after injection, and the hole was filled with bone wax. Finally, we irrigated the wound with saline, closed it with silk sutures, and dusted the wound with penicillin powder.

### 2.3. Radiological and Histochemical Analysis of Bone

To determine whether the tibial bone was destroyed by tumor inoculation, the rats underwent X-rays on the 21st day after the operation. Moreover, to visualize histologic changes in the bone marrow and tumor, we decalcified the right tibia in 10% EDTA for two weeks prior to paraffin embedding. The tibia was abscised 4 μm below the frontal plane and then stained with eosin and hematoxylin. An inverted microscope was used to take photographs.

### 2.4. Pain Behavior Test

The subjects were placed in a 22 cm high plastic cage with the bottom made from wire mesh. Each grid measured 0.5 cm by 0.5 cm. Then, the up-and-down method was adopted to assess the MWT [19]. Briefly, after 15–20 min of adaptation, von Frey filaments varying from 0.4 g to 15 g were adopted for the pain stimuli. The test was initiated by applying 2.0 g of hair vertical to the surface of the paw until the filament bent, and then holding it there for 6–8 s. A positive response was noted as a sudden withdrawal of the paw. The next weaker stimulus was presented once there was a positive response. Conversely, a stronger stimulus was presented if there was a negative response. Six stimuli were presented following the first change in response. Then, the 50% mechanical withdrawal threshold (MWT) was determined as previously described [19].

### 2.5. Experimental Groups and Treatment

To probe the contribution of VGSCs in CIBP, the rats were randomized into two groups, namely, the sham group and CIBP group. Then, the gene expression profiles were assessed by transcriptome sequencing.

To verify the role of Na_v_1.6-expressing neurons in CIBP, 14 rats were randomly assigned to each of six groups: the sham, CIBP, negative control lentivirus, shNa_v_1.6#1, shNa_v_1.6#2, and shNa_v_1.6#3 groups. Negative control shRNA (10 μL) or shRNA targeting Na_v_1.6 (2 × 10^6^ TU/10 μL) was intrathecally injected into the rats 7–14 days after establishing the model.

To further explore whether activation of the MAPK signaling induces CIBP by regulating the expression of Na_v_1.6, the p38 MAPK inhibitor SB203580 (2 μg/10 μL, Selleck), the Jun N-terminal kinase (JNK) inhibitor SP600125 (20 μg/10 μL, Selleck), the extracellular signal-regulated kinase (ERK) inhibitor U0126 (10 μg/10 μL, Selleck) or vehicle (4% DMSO + 40% PEG300 + 5% Tween80 + ddH_2_O, 10 μL Med Chem Express) were intrathecally injected in the rats 12–14 days after establishing the model. Pain behaviors were evaluated 2, 4, 6, and 8 h after drug application.

### 2.6. DRG Collection

In summary, the rats were anesthetized by injecting pentobarbital sodium (50 mg/kg) intraperitoneally. Then, a skin incision was made, and the muscles were gently separated from the spine. The spine was cut at the hip level and dissected on ice. The spinous processes and transverse processes of the spine were exposed, the surrounding connective tissue was completely removed, and the spinal cord was slowly lifted. The enlarged DRGs (L4-L6) in the intervertebral foramen were attached to the posterior root, which was approximately 1 mm in length, light yellow, and oval, and had a smooth surface. The enlarged part (DRG) was removed from the spinal nerve posterior root. Then, the DRGs, were placed in sterilized frozen storage tubes after being washed in normal saline and kept in a −80 °C freezer.

### 2.7. RNA-seq and Bioinformatics Analysis

Twenty female SD rats were randomly divided into two groups (the sham and CIBP groups). Three replicate samples from each group were sent for mRNA sequencing analysis, which was carried out in collaboration with Gene Denovo Biotechnology Co. (Guangzhou, China). The total RNA of the DRGs was isolated using an Invitrogen TRIzol reagent kit. RNA quality was assessed using an Agilent 2100 Bioanalyzer. To further verify the RNA integrity, agar gel electrophoresis was utilized. An oligo (dT) bead enrichment procedure was conducted after the total RNA extraction. Reverse transcription of the target mRNA fragments into ds-cDNA (double-stranded cDNA) was performed suing random primers. Then, the library was constructed from cDNA fragments that had been purified, repaired, poly(A)-tailed, and ligated. The Illumina NovaSeq 6000 platform was employed to sequence the library. Differential expression analysis of the RNA-seq data between two different groups was performed with DESeq2 software. Significantly differentially expressed genes/transcripts were identified as having an FDR below 0.05 and absolute fold change ≥ 1.5. GO and KEGG pathway enrichment analyses were performed.

### 2.8. Total RNA Extraction and RT-qPCR

TRIzol reagent was used to extract total RNA from the rat DRG samples. HiScript III RT SuperMix for qPCR (+gDNA wiper) (Vazyme Biotech Co., Ltd., Nanjing, China) was used to synthesize cDNA following the manufacturer’s protocol. RT-qPCR proceeded using a 2× ChamQ Universal SYBR qPCR Master Mix (Vazyme Biotech Co., Ltd., Nanjing, China) (10 μL), forward primer (10 μΜ/0.4 μL), reverse primer (10 μΜ/0.4 μL), cDNA (2 μL), and nuclease-free water (up to 20 μL). The target gene expression was normalized to the GAPDH expression following the 2^−ΔΔ^Ct formula. The primer sets were synthesized by Fuzhou Sunya Biotechnology (China), and the primer sequences for the target gene and GAPDH are shown in Table 1.

### 2.9. Western Blotting

DRG samples from lumbar vertebrae 4–6 were obtained and stored at −80 °C. The samples were lysed in a lysis buffer containing 1% protease inhibitor and PMSF. Then, the samples were centrifuged at 12,000× *g* for 15 min at 4 °C to obtain the supernatants. Equal total protein (30 μg) was separated by 10% SDS-PAGE and transferred to a PVDF membrane. The membrane was blocked with 5% fat-free milk for 1 h at room temperature (RT). Then, the cells were incubated with primary antibodies against rabbit anti-Na_v_1.6 (1:1000; Abcam, Cambridge, UK), rabbit anti-p38, rabbit anti-p-p38, rabbit anti-JNK, rabbit anti-p-JNK, mouse anti-ERK, and rabbit anti-p-ERK (1:1000; CST, Boston, MA, USA) overnight at 4 °C, followed by a secondary antibody (1:10,000; Bioworld Technology, St. Louis Park, MN, USA). For the load control, a rat antibody against β-actin (1:10,000; CST, Boston, MA, USA) was employed.

### 2.10. Immunofluorescence

The rats were anesthetized with 2% isoflurane and perfused with 4% paraformaldehyde on the 21st day after establishing the model. The L4-6 DRGs were harvested, fixed with 4% paraformaldehyde overnight, dehydrated, and embedded in paraffin. Sections were cut to 7 μm thickness and mounted on glass slides. Afterwards, the tissue sections were removed from paraffin. Sections were blocked in 5% BSA for 20 min, followed by incubation with rabbit anti-Na_v_1.6 antibody (1:100 Abcam, Cambridge, UK) at 4 °C overnight. Then, fluorescein isothiocyanate (FITC)-conjugated AffiniPure goat anti-rabbit IgG (H+L) (1:100, Proteintech Group, Inc., Wuhan, China) was added to the slides for 1 h at 37 °C. Images were taken with a microscope camera (Q-imaging, Shanghai, China) attached to an IX51 microscope (Olympus, Tokyo, Japan) after the slides were rinsed and sealed.

### 2.11. Intrathecal Catheter Implantation

A previously described method was adopted in this research with some modifications. To summarize, the rats were anesthetized by 1–2% isoflurane, and a polyethylene-10 catheter filled with normal saline was inserted into the subarachnoid space at the L5/L6 level. Then, the tip of the catheter was placed at the L5 level. The outer end of the catheter was subcutaneously excavated in the head and fixed to the skin with a silk ligation to avoid being bitten by the rat. More than 4 cm of the catheter was left outside the skin on the nape of the neck. Three days after operation, 10 μL of 2% lidocaine was infected into each rat through the catheter. Animals not temporarily paralyzed in the posterior limbs as a result of lidocaine injection were excluded from our study.

### 2.12. Construction of Na_v_1.6 shRNA-Expressing Lentivirus

A sequence-specific guide RNA targeting the gene encoding Na_v_1.6, which was used to knockdown Na_v_1.6, was designed by and purchased from Gene Chem. A negative control shRNA was defined as a lentivirus targeting the sequence without significant homology to any known human genes (Gene Chem, Shanghai, China). Lentiviral vectors were applied to deliver shNa_v_1.6 to DRG neurons in vivo. The sequences of the shRNA targeting the Na_v_1.6 gene and the negative control shRNA were as follows:

shNa_v_1.6#1: CGCCTTATGACCCAGGACTAT
shNa_v_1.6#2: GGCCATGTGCCTCATTGTCTT
shNa_v_1.6#3: AAGCAGATGGAGAACATTCTT
negative control shRNA: TTCTCCGAACGTGTCACGT

### 2.13. Statistical Analyses

Statistical analysis was performed with GraphPad Prism 8. The data are presented as the mean ± SEM. Differences between two groups were analyzed by unpaired Student’s *t* test. Differences among three groups were analyzed by one-way analysis of variance (ANOVA) followed by Tukey’s post hoc test. The MWT results were analyzed by two-way repeated-measures ANOVA followed by Bonferroni’s post hoc test. *p* < 0.05 was considered statistically significant.

## 3. Results

### 3.1. Successful Induction of CIBP in Rats

**Figure 1:**
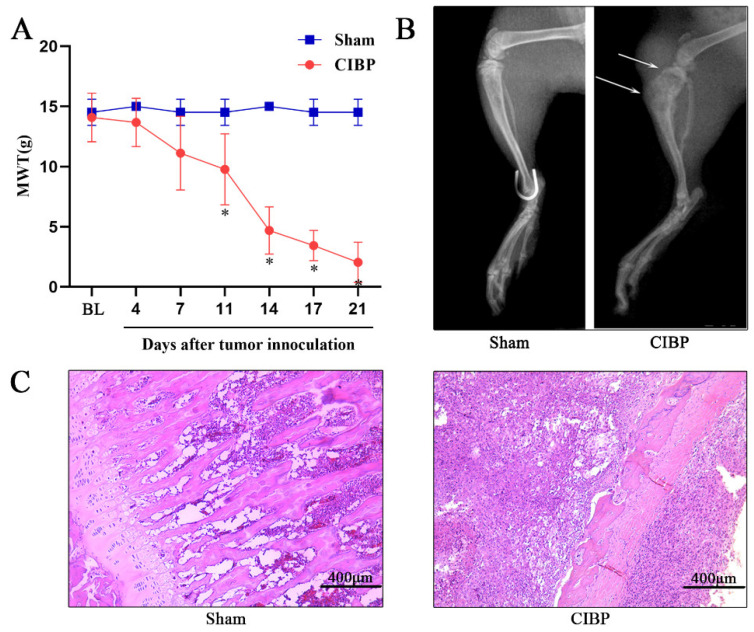
Transplantation of MRMT-1 carcinoma cells induces hyperalgesia and bone destruction. (**A**) Mechanical hypersensitivity was observed beginning 11 days after intratibial inoculation of MRMT-1 cells and lasted until the 21st day, while the MWTs of the sham group remained stable. The data are presented as the mean ± SD (* *p* < 0.05). (**B**) X-ray examination of the right hind paw showing bone destruction (marked by the white arrow). (**C**) Microscopic examination of tibia slices by HE staining showing that the medullary cavity was filled with carcinoma cells instead of bone marrow cells. MWT, mechanical withdrawal threshold; BL, baseline; Sham, sham-operated group; CIBP, cancer-induced bone pain group.

Pain caused by MRMT-1 cancer cell inoculation was evaluated by measuring the 50% mechanical withdrawal threshold (MWT) using von Frey hairs. The baseline MWT of the sham group were similar to those of the CIBP group (*p* > 0.05). The MWT of rats in the CIBP group began to decrease from day 11 to day 21 after tumor cell inoculation (*p* < 0.05) (Figure 1A). X-ray imaging revealed that the cortical bone had a complete structure and was continuous in the sham group. However, the tibia of rats in the CIBP group lacked trabecular bone and continuous cortical bone and showed obvious swelling of the soft tissue around the bone (Figure 1B). HE staining showed the presence of a large number of large and strongly stained, dual-nuclei tumor cells in the bone marrow cavity and severe damage to the bone structure (Figure 1C). 

### 3.2. Changes in Gene Expression Profiles Assessed by RNA Sequencing (RNA-seq) and Gene Ontology (GO) and KEGG Pathway Enrichment Analysis

**Figure 2:**
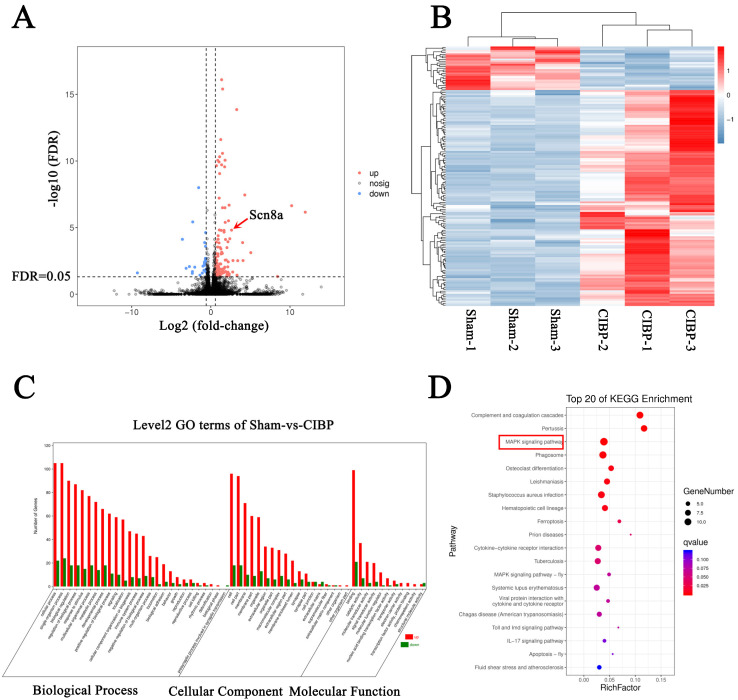
Significant changes in mRNA expression in the DRGs of rats with CIBP identified by high-throughput transcriptomic sequencing. (**A**) Volcano plot of DEmRNAs. The vertical line indicates a 1.5-fold change in expression (upregulation or downregulation); the horizontal line indicates thresholds for 5% FDR. The red dots indicate upregulated mRNAs, and the blue dots indicate downregulated mRNAs. (**B**) Hierarchical cluster analysis of DEmRNAs. The color (from blue to red) represents the level of gene expression (log10 FPKM) from low to high, indicating the downregulation and upregulation of the expression. (**C**) Top 25 GO enrichment terms in the three groups, i.e., biological process, cellular component, and molecule function, for the DEmRNAs according to the *p* value. (**D**) Top 20 pathways in which the DEmRNAs were enriched according to KEGG pathway enrichment analysis; the size of the bubble indicates the number of enriched mRNAs, the color indicates the *p* value, the red frame highlights the MAPK signaling pathway, which may be associated with CIBP.

The mRNA expression patterns in the L4-L6 DRGs of rats with CIBP and sham rats were evaluated. Compared with the sham group, the expression of 124 genes was upregulated and that of 25 genes was downregulated in the CIBP group, as shown in Figure 2A,B. The expression of *Scn8a* (which encodes Na_v_1.6), one of the main VGSC subtypes in rat DRG neurons, was shown to be markedly upregulated in the CIBP group (Figure 2A).

To further study the function of the DEmRNAs, we then analyzed GO and KEGG pathway enrichment. GO analysis demonstrated that the DEmRNAs were enriched in the biological process terms regulation of the biological process and signaling, the cellular component terms membrane and macromolecular complex, and the molecular function term nucleic acid-binding transcription factor activity and transporter activity (Figure 2C). The KEGG analysis showed that the DEmRNAs were mainly enriched in complement and coagulation cascades, the MAPK signaling pathway, the cytokine–cytokine receptor interaction, the IL-17 signaling pathway, etc. (Figure 2D).

The top 10 upregulated and downregulated genes are listed in Supplemental Table S1. To test the reliability of the sequencing data, the levels of 6 DEmRNAs (the top three upregulated and three downregulated mRNAs) in another independent sample were examined by real-time quantitative polymerase chain reaction (RT-qPCR). As shown in Supplemental Figure S1, the mRNA expression of *LOC100911356* and *Ndst2* was upregulated in the CIBP group, and the expression of *Prss29, Epyc, and Fcrla* was significantly downregulated, which is consistent with the RNA-seq results.

### 3.3. Upregulation of Na_v_1.6 Expression in the DRGs in Rats with CIBP

**Figure 3:**
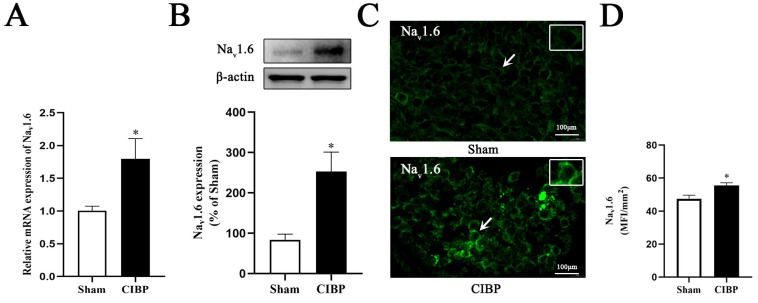
The expression of Na_v_1.6 is increased in the DRG of rats with CIBP. (**A**) The Na_v_1.6 mRNA levels were increased in rats with CIBP (* *p* < 0.05). (**B**) The Na_v_1.6 protein levels were increased in rats with CIBP. (**C**) The ratio of Na_v_1.6-positive neurons was significantly increased in the rats with CIBP (* *p* < 0.05). Arrows denoted the distribution and expression of Na_v_1.6 in single neuron in Sham and CIBP group. (**D**) Relative fluorescence intensity of Na_v_1.6 in DRG was increased in CIBP group. ( * *p* < 0.05).

To confirm the change in Na_v_1.6 expression in the DRGs of rats with CIBP, we assessed the Na_v_1.6 expression levels 21 days after MRMT-1 cell injection. The mRNA and protein levels of Na_v_1.6 were significantly increased (Figure 3A,B). Immunofluorescence staining also showed that the number of Na_v_1.6-positive neurons in the DRGs of rats with CIBP was increased (Figure 3C,D).

### 3.4. Na_v_1.6 Gene Knockdown Reduces Mechanical Hypersensitivity in Rats with CIBP

**Figure 4:**
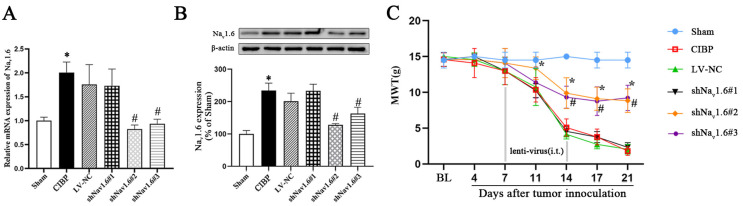
Nav1.6 knockdown reduces mechanical hypersensitivity induced by CIBP. (**A**) RT-PCR and (**B**) Western blotting showed that lentiviruses expression shRNA targeting Na_v_1.6 sequence 2 (shNa_v_1.6#2 group) and sequence 3 (shNa_v_1.6#3 group) significantly reduced the expression level of Na_v_1.6. (**C**) Dynamic changes in mechanical sensitivity after lentivirus treatment. The MWTs of rats in the shNa_v_1.6#2 group and shNa_v_1.6#3 groups decreased more slowly than the MWT of rats in the CIBP group (* *p* < 0.05, compared with the sham group; ^#^ *p* < 0.05 compared with the CIBP group).

To examine the effect of Na_v_1.6 in rats with CIBP, lentivirus expressing one of three shRNA targeting specific regions of the Na_v_1.6 sequence (2 × 10^6^ TU/10 μL) or a control nontargeting shRNA (10 μL) was intrathecally injected 7–14 days after the operation. We found the mRNA and protein levels of Na_v_1.6 were markedly reduced in the rats that received lentivirus targeting Na_v_1.6 sequence 2 (shNa_v_1.6#2 group) and sequence 3 (shNa_v_1.6#3 group) compared with the rats that received control shRNA (*p* < 0.05 vs. the CIBP group, Figure 4A,B). Then, the MWT of each group of rats was measured (Figure 4C). The baseline MWTs were similar between the groups (*p* > 0.05). The results showed that the MWT of the rats in the shNa_v_1.6#2 group and shNa_v_1.6#3 groups decreased more slowly than that of the rats in the CIBP group (11 d, 14 d, 17 d, and 21 d after the operation, *p* < 0.05, the shNa_v_1.6#2 group vs. the CIBP group; 14 d, 17 d, and 21 d after the operation, *p* < 0.05, the shNa_v_1.6#3 group vs. the CIBP group).

### 3.5. p38 MAPK Inhibition Significantly Increases the Mechanical Pain Threshold in Rats with CIBP

**Figure 5:**
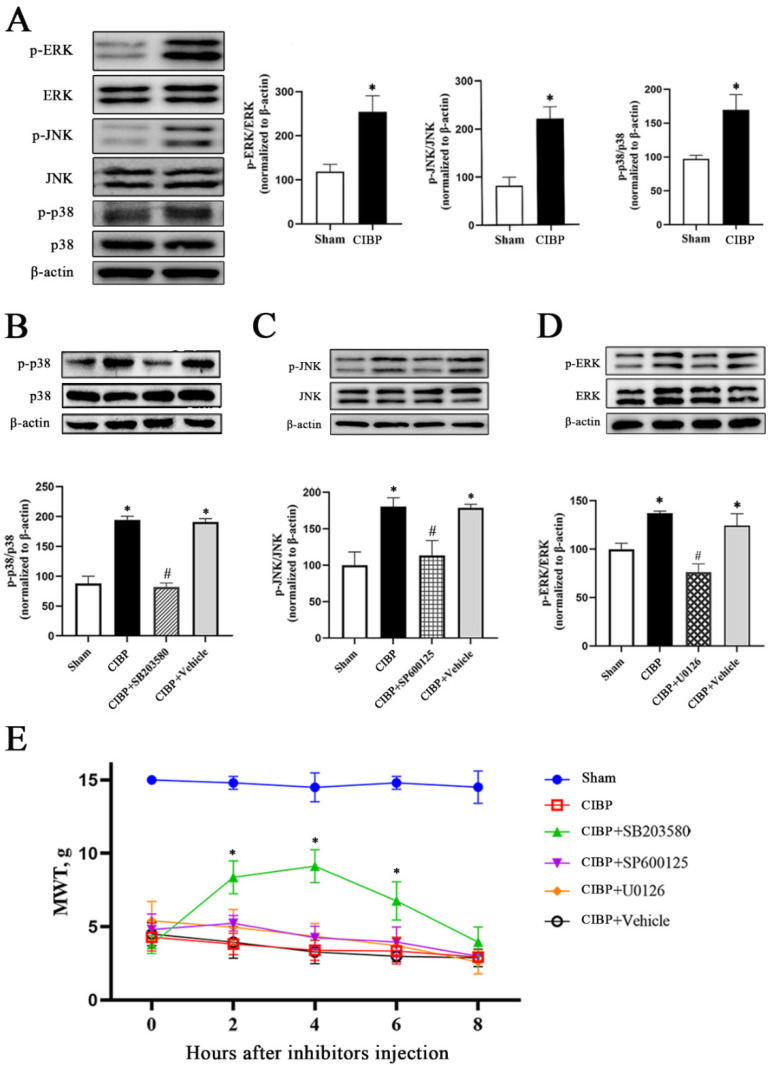
Inhibition of p38 MAPK increases mechanical sensitivity in rats with CIBP. (**A**) Western blotting showed that the phosphorylation of p38 MAPK/JNK/ERK (p-p38, p-JNK, and p-ERK levels) in the DRGs was significantly increased in rats with CIBP (* *p* < 0.05 vs. the sham group). (**B**) Inhibition of the expression of p-p38, (**C**) p-JNK, and (**D**) p-ERK. * *p* < 0.05 compared with the sham group; ^#^ *p* < 0.05 compared with rats in the CIBP group. (**E**) Dynamic changes in mechanical sensitivity after inhibitor treatment. Inhibition of p38 increased the mechanical pain threshold (* *p* < 0.05 compared with the CIBP group).

To explore whether the MAPK pathway was involved in CIBP, the phosphorylation of p38 (p-p38), JNK (p-JNK), and ERK (P-ERK) in the DRGs was evaluated by Western blotting. Then, inhibitors of p38 (SB203580), JNK (SP100125), and ERK1/2 (U0126) were injected intrathecally from day 12 to day 14 after operation. We found that the phosphorylation levels of p38, JNK, and ERK were markedly increased in rats with CIBP, as shown in Figure 5A (*p* < 0.05 vs. the sham group), while intrathecal injection of SB203580, SP100125, and U0126 effectively inhibited CIBP-induced p38, JNK, and ERK phosphorylation in the DRGs, respectively (Figure 5B–D; *p* < 0.05 vs. the CIBP group). Then, we measured the changes in the MWTs of rats before inhibitor administration and 2, 4, 6, and 8 h after administration with von Frey filaments. The results showed that only the MWT of the CIBP + SB203580 group increased significantly (2, 4, and 6 h after administration; *p* < 0.05 vs. the CIBP group), while there was no significant difference in MWT between the CIBP + SP600125 group or CIBP+U0126 group and CIBP group (Figure 5E, *p* > 0.05 vs. the CIBP group), indicating that p38 MAPK, but not JNK or ERK, was involved in CIBP in rats.

### 3.6. p38 MAPK Works Upstream of Na_v_1.6 in Rats with CIBP

**Figure 6:**
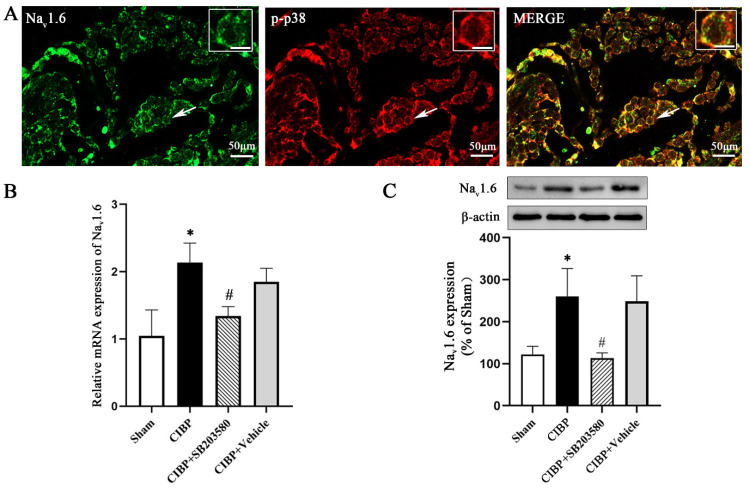
p-p38 regulates Na_v_1.6 expression in DRG neurons. (**A**) Immunofluorescence staining of Na_v_1.6 and p-p38. Na_v_1.6 colocalized with p-p38 in the DRG neurons of CIBP rats. Scale bar is 50 μm in lower magnifications and 10 μm in higher magnifications. (**B**) Na_v_1.6 mRNA expression was decreased after intrathecal injection of a p38 inhibitor (SB203580). (**C**) Na_v_1.6 protein expression was decreased after intrathecal injection of a p38 inhibitor (SB203580) (* *p* < 0.05 vs. the sham group, ^#^ *p* < 0.05 vs. the CIBP group).

To further clarify whether p38 MAPK regulates the expression of Na_v_1.6 and contributes to the initiation of CIBP, we first performed double immunofluorescence to examine the localization of Na_v_1.6 and p-p38 in DRG neurons. The results showed that p-p38 and Na_v_1.6 were colocalized in the DRGs (Figure 6A). Then, we evaluated the expression level of Na_v_1.6 in the DRGs after the intrathecal application of a p38 inhibitor. As shown in Figure 6B,C, the mRNA and protein levels of Na_v_1.6 in the CIBP + SB203580 group were markedly reduced when compared with the CIBP group (*p* < 0.05).

## 4. Discussion

In the present study, CIBP was constructed by injecting MRMT-1 breast cancer cells into the right tibias of female SD rats. RNA-seq of DRG neurons from CIBP rats showed that Na_v_1.6 mRNA was highly upregulated. Moreover, intrathecal injection of lentivirus expressing Na_v_1.6-targeted shRNA efficiently relieved CIBP. Moreover, inhibition of the p38 MAPK pathway significantly reduced Na_v_1.6 expression and attenuated pain behavior. 

CIBP is one of most common complications of cancer [20]. Approximately 75% of patients with bone metastasis experience moderate or severe pain, severely affecting patients’ quality of life [21]. Therefore, exploring the mechanism of CIBP to provide insight into potential treatment options is very important. Animal models of CIBP established by intratibial injection of tumor cells have been widely studied [22]. The incidence of breast cancer bone metastasis is approximately 70%, and global cancer statistics from 2020 showed that breast cancer has become the most common malignant tumor worldwide [23]. With the increasing number of breast cancer patients with bone pain, an animal model of CIBP has been adopted using breast cancer cells. Consistent with other studies, time-dependent mechanical hypersensitivity was observed on days 11–21 after MRMT-1 cell injection [24].

VGSCs in the DRGs are fundamental for determining the excitability of primary sensory neurons, resulting in pain sensations. Therefore, targeting VGSCs on peripheral sensory neurons may become an important strategy for treating pain. However, studies on the role of VGSCs in CIBP are limited and controversial. Our RNA-seq data showed that compared with that of other sodium channel subtypes, the Na_v_1.6 expression was significantly upregulated in the DRGs of rats with CIBP. Na_v_1.6 is a TTX-s VGSC with fast kinetics [25]. These channels are characterized by slow inactivation and fast repriming, and mediate persistent and resurgent currents in DRG neurons [26]. Tanaka found a case of trigeminal neuralgia with a function gain mutation in Na_v_1.6 [13]. Several studies have shown that Na_v_1.6 is involved in neuropathic pain caused by chemotherapy drugs (such as vincristine and oxaliplatin) or SNL [27,28,29]. Consistent with these studies, the Na_v_1.6 mRNA and protein expression were markedly upregulated in the DRGs of rats with CIBP on the 21st day after operation in our research. Moreover, knocking down Na_v_1.6 in the DRGs by intrathecal injection of an shRNA-expressing lentivirus from day 7 to day 14 after the operation significantly alleviated CIBP in rats. 

The MAPK family is a family of serine/threonine protein kinases that includes ERK, JNK, and p38 MAPK. A variety of extracellular stimuli, such as inflammatory factors and cytokines, can activate the MAPK pathway [30,31]. A growing body of research has indicated that the MAPK pathway plays an important role in chronic pain [32]. However, there have been few reports on the activation of MAPK in the DRGs of rats with CIBP. In this study, KEGG analysis of the DEmRNAs revealed that MAPK pathway was involved in CIBP. ERK, p38, and JNK phosphorylation were increased in the DRGs of rats with CIBP 21 days after operation. In addition, intrathecal injection of specific inhibitors of JNK, ERK, and p38 12–14 days after operation significantly reduced p-JNK, p-ERK, and p-p38 expression. However, only the p38 inhibitor SB203580 alleviated hyperalgesia in rats with bone cancer. In previous studies, p38 phosphorylation in DRGs was observed to increase in a chronic compression injury induced neuropathic pain [33]. p-ERK expression in the spinal dorsal horn was enhanced in a neuropathic pain induced by SNL [34]. After chronic constriction injury (CCI), damage to DRG neurons induces an increase in p-ERK expression, and continuous intrathecal injection of the ERK upstream MEK1 inhibitor U0126 inhibits mechanical allodynia caused by CCI [35]. It differs from these findings in neuropathic pain, where our results demonstrated that activation of the p38 MAPK pathway in the DRGs, but not the JNK and ERK pathways, played a key role in CIBP. The p38 MAPK signaling in DRGs can be activated by both inflammatory factors released by tumor cells and immune cells associated with tumors and direct nerve injury resulting from tumor cell invasion. 

There is ample evidence that activating the p38 MAPK pathway can regulate intracellular responses, drive intracellular transcription and posttranslational processes, and upregulate the expression of certain pain-related genes [30,36,37]. However, whether p38 MAPK can upregulate Na_v_1.6 expression in the DRGs to contribute to CIBP has not yet been reported. In our experiment, we observed that the colocalization of p38 with Na_v_1.6 in the DRGs. Furthermore, p38 MAPK was activated by phosphorylation, followed by upregulation of Na_v_1.6 in the DRGs of CIBP rats, and the intrathecal application of a p38 inhibitor significantly reduced Na_v_1.6 mRNA and protein expression in the DRGs and alleviated mechanical hyperalgesia in rats with CIBP. Wittmack et al. discovered that p38 MAPK colocalized with Na_v_1.6 in the brains of rats, and proposed for the first time that in vitro domain L1 of Na_v_1.6 at serine 553 was phosphorylated following the activation of p38α, reducing the current density of Na_v_1.6 without changing the channel gating characteristics [38]. Chen and other researchers found that long-term TNF-α stimulation (8–24 h) can upregulate the mRNA and protein expression of Na_v_1.6 in vitro in mouse cortical neurons [39]. In short, these findings show that the interplay between p38 MAPK and VGSCs, especially Na_v_1.6, may be related to the exposure duration and neuron type.

A special tumor microenvironment that contains a large amount of acidic substances, inflammatory mediators, and cytokines is created when tumor cells metastasize to bone [40]. These substances are transported to the DRG. It is possible that prolonged inflammation causes activation of the p38 MAPK signaling pathway [41], and promotes transcription factor binding to the corresponding regions of the Na_v_1.6 sequence, which may increase the transcription and translation of the Na_v_1.6 protein and contribute CIBP. Further studies of the mechanism of CIBP and the role of MAPK pathway-regulated VGSCs are needed to identify potential targets for the treatment of CIBP.

In conclusion, the present study showed that upregulation of Na_v_1.6 expression mediated by p38 MAPK pathway activation in the DRGs of rats contributes to CIBP.

## Figures and Tables

**Table 1:** Primer sequences.

Genes	Sequences (5′–3′)
LOC100911356	F-AGTATCAGCAGCAGCACAGT
	R-AGGGCATCCAGGTCAAAGTT
Ndst2	F-CGCTCCTCTGCTACATCTCA
	R-GCTCATAGGTGCTGTGATTGG
Cct8l1	F-GCTGACTACTGTGGCGTCAT
	R-AGGTGCAATCTCACGTTCC
Prss29	F-CCCTTTGTGAGAAGCTGTATCG
	R-GAGTCACCATAGCAGGAGTCT
Epyc	F-GTTCGTAAGGCGCTAGAGGA
	R-TACCAATGGGCAAACGAGGT
Fcrla	F-CCGGATAAACCGCCTTCTCA
	R-TTTGTAGTGGCAGGCTTCCG
Scn8a	F-ACTGGACGATACCAGCTCCT
	R-TTCCTCGATGTTGACCTGGC
GAPDH	F-ACGGCAAGTTCAACGGCACAG
	R-GAAGACGCCAGTAGACTCCACGAC

## Data Availability

Data are available from the corresponding author upon specific request.

## Supplementary Materials

The following supporting information can be downloaded at: https://www.mdpi.com/article/10.3390/cells11213375/s1, Table S1: The detailed information of top 10 up-regulated and top 10 down-regulated mRNAs; Figure S1: The mRNA expression of LOC100911356 and Ndst2 was upregulated in the CIBP group, and the expression of Prss29, Epyc, and Fcrla was significantly downregulated.
