# Supplementary material for: Upregulation of Nav1.6 Mediated by the p38 MAPK Pathway in the Dorsal Root Ganglia Contributes to Cancer-Induced Bone Pain in Rats
Source: Cells. 2022 Oct 26;11(21):3375. doi: 10.3390/cells11213375 (PMC9654392; doi:10.3390/cells11213375)
Supplement: Supplementary file 1 [file cells-11-03375-s001.zip › cells-1912516-supplementary.pdf]

Supplemental Table S1 The detailed information of top 10 up-regulated and top 10 down-regulated mRNAs.

Gene name	description	Log_2FC	FDR
**Up-regulated**			
LOC100911356	peroxisomal biogenesis factor 19-like	11.9403136	7.05E-07
Ndst2	N-deacetylase and N-sulfotransferase 2	10.20864081	2.30E-07
Cct8l1	chaperonin containing TCP1, subunit 8	8.423466121	0.04638744
Alox15	arachidonate 15-lipoxygenase	5.055365237	0.000760195
Ptprh	protein tyrosine phosphatase, receptor	4.297680549	3.59E-08
Vtcn1	V-set domain containing T cell activation	4.075288127	0.002951496
Stac2	SH3 and cysteine rich domain 2	4.025355574	0.000135587
Pcdh20	protocadherin 20	3.40599236	0.00307595
S1pr5	sphingosine-1-phosphate receptor 5	3.315641832	0.037585937
Scn8a	sodium voltage-gated channel alpha	2.402484448	6.80E-05
**Down-regulated**			
Prss29	protease, serine, 29	9.276124405	0.025252181
Epyc	epiphycan	3.584962501	7.65E-05
Fcrla	Fc receptor-like A	3.115477217	0.011394184
Mepce	methylphosphate capping enzyme	2.696607857	0.008398052
Cd79b	CD79b molecule	2.340624189	0.027160399
Rag1	recombination activating 1	2.321928095	0.019879991
Nppb	natriuretic peptide B	2.288840707	3.83E-06
Spib	Spi-B transcription factor	2.037474705	0.009205265
LOC100360449	ribosomal protein L9-like	1.547742115	1.01E-08
Pgam2	phosphoglycerate mutase 2	1.381346305	0.036898829

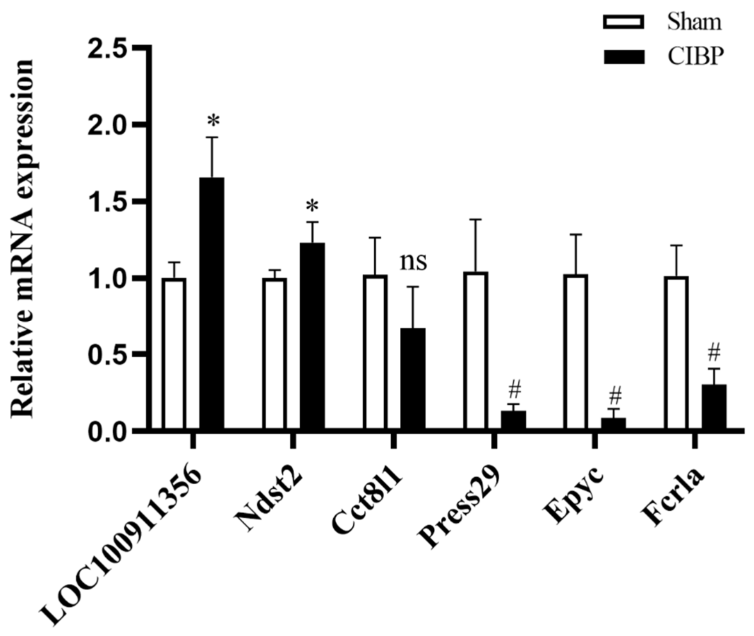

Supplemental Figure S1: The mRNA expression of LOC100911356 and Ndst2 was upregulated in the CIBP group, and the expression of Prss29, Epyc, and Fcrla was significantly downregulated, which is consistent with the RNA-seq results.
